# Coil-assisted retrograde transvenous obliteration (CARTO) for the treatment of gastric varices via a single access route using steerable microcatheter: a case report

**DOI:** 10.1186/s42155-020-00124-3

**Published:** 2020-06-14

**Authors:** Kanako Terada, Takahiro Ogi, Norihide Yoneda, Akira Yokka, Takumi Sugiura, Wataru Koda, Satoshi Kobayashi, Toshifumi Gabata

**Affiliations:** 1 Department of Radiology, Kanazawa University Graduate School of Medical Sciences, 13-1 Takaramachi, Kanazawa, Ishikawa 920-8640 Japan; 2 Department of Quantum Medical Imaging, Kanazawa University Graduate School of Medical Sciences, 13-1 Takaramachi, Kanazawa, Ishikawa 920-8640 Japan

**Keywords:** Gastric varices, BRTO, CARTO, Steerable microcatheter

## Abstract

**Background:**

Coil-assisted retrograde transvenous obliteration (CARTO) is a modified balloon-occluded retrograde transvenous obliteration (BRTO) technique using coils instead of an indwelling balloon. A method involving two microcatheter systems forming a double access route has been reported. We report a case of CARTO using a steerable microcatheter to successfully treat gastric varices (GV).

**Case presentation:**

A 79-year-old man was admitted for treatment of intractable GV due to liver cirrhosis. The GV were drained mainly into the left inferior phrenic vein, not the usual gastrorenal shunt. Introducing the balloon catheter to the left inferior phrenic vein was difficult due to mild stenosis between the inferior vena cava and inferior phrenic vein and the shunt angle. A CARTO technique was performed with 5% ethanolamine oleate with iopamidol from a single access route by inverting the steerable microcatheter distal to the coil placement site.

**Conclusion:**

CARTO has advantages in cases where performing BRTO is difficult. Using a steerable microcatheter simplifies the procedure by reducing the required access routes in CARTO.

## Introduction

Balloon-occluded retrograde transvenous obliteration (BRTO) was first described by Kanagawa et al. in 1996 (Kanagawa et al. 1996). It is a well-described and established procedure for the treatment of gastric varices (GV), and there have been a number of reports on the long-term efficacy of this technique (Ninoi et al. 2005). However, the placement of an indwelling inflated balloon requires increased procedural time and extensive monitoring, and balloon rupture may cause adverse events. Therefore, modified BRTO techniques have been developed (Gwon et al. 2013; Kim et al. 2018; Kobayakawa et al. 2019; Lee and Jeon 2018) . Lee et al. introduced coil-assisted retrograde transvenous obliteration (CARTO) in 2012 and reported on 20 cases of the procedure in 2014 (Lee et al. 2014). This technique is a modified BRTO technique which uses coils instead of an indwelling balloon and is advantageous if the shunt is not conducive to balloon placement. The use of two microcatheter systems, one each for coil deployment and gelfoam injection, from a double access route has been reported in CARTO.

The steerable catheter has the advantages of selective catheterization of acute-angle branching vessels and the possibility of compact coil packing with intentional folding by the bendable tip of the catheter (Soyama et al. 2017; Inaba et al. 2019) . Here, we report a successful case of CARTO performed for the treatment of intractable GV via a single access route with a steerable microcatheter.

## Case report

**Fig. 1:**
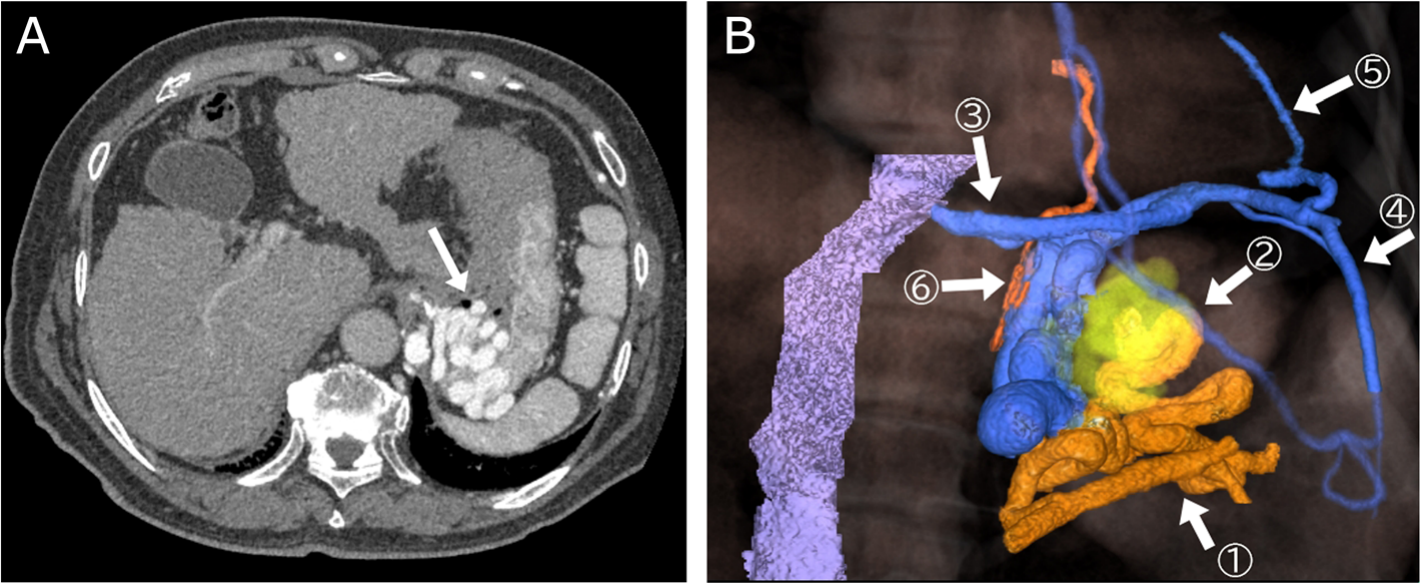
**a** Representative computed tomography during arterial portography image showing gastric varices (white arrow) before treatment. **b** Three-dimensional volume rendered images of gastric varices reconstructed from computed tomography during arterial portography. The main feeding vessels of gastric varices are the posterior and short gastric vein (①). The varices (②) drained mainly into the left inferior phrenic vein (③), the gastrorenal shunt was absent. The left internal thoracic vein (④), pericardial phrenic vein (⑤), and left inferior pulmonary vein (⑥, which is from the outflow shunt) were other drainage veins. Mild stenosis is observed between the inferior vena cava and left inferior phrenic vein.

A 79-year-old man was admitted to our hospital for treatment of intractable GV due to liver cirrhosis prior to surgery for hepatocellular carcinoma (HCC). Endoscopic sclerotherapy was performed 4 years previously, but GV were worsening. Based on endoscopic finding, the classification of GV was F3, Lg-CF, and white (Cw). The Child-Pugh score was 6 (class A). Laboratory data showed: platelets, 120,000/μl; prothrombin-international normalized ratio, 1.12; creatinine, 1.18 mg/dl. Angio-computed tomography (CT) was performed prior to treatment to evaluate the hemodynamics of GV and work up for HCC. CT during arterial portography (CTAP) was performed via the superior mesenteric artery, and the scanning was started 25 s after the infusion of 50 ml of iohexol (350mgI/ml) at a rate of 1.8 ml/sec was started. From CTAP, GV were revealed to drain mainly into the left inferior phrenic vein, and the gastrorenal shunt was absent. The main feeding vessels of GV were the posterior and short gastric vein. The left internal thoracic vein, pericardial phrenic vein, and left inferior pulmonary vein were observed to be collateral veins (Fig. 1). We initially planned BRTO from the left inferior phrenic vein. Single right femoral venous access was achieved and the guidewire (0.032 or 0.035 in., Radifocus, Terumo, Tokyo, Japan) was introduced into the inferior phrenic vein. However, mild stenosis between the inferior vena cava (IVC) and inferior phrenic vein, and the shunt angle, prevented introduction of the 6 Fr balloon catheter (Selecon MP Catheter II, Terumo, Tokyo, Japan). Therefore, CARTO was planned and attempted from the same single route.

**Fig. 2:**
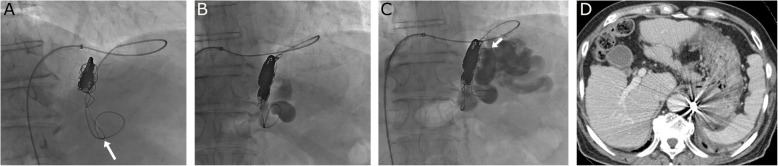
**a** The steerable catheter was inverted from distal to the coil embolization site (white arrow). Detachable coils were deployed using a 1.9 Fr micro catheter through the inverted steerable catheter. **b** Complete coil occlusion of the outflow shunt was confirmed and the inferior phrenic vein was not visualized. **c** We gradually injected 5% ethanolamine oleate with iopamidol (total 24 ml) through the microcatheter (white arrow) to embolize up to the coil in outflow shunt, varices, other minor collaterals. **d** Contrast enhanced computed tomography performed 1 week after coil-assisted retrograde transvenous obliteration revealed that complete occlusion of the gastric varices (white arrow) was achieved (portal venous phase)

A 7 Fr Flexor Ansel guiding sheath (Cook Medical, Bloomington, IN, USA) was placed in the IVC before the stenosis site, using 0.035 in. guidewire and 4 Fr non tapered angle catheter (Terumo, Tokyo, Japan). The left inferior phrenic vein was successfully accessed using 4 Fr catheter and 2.9 Fr steerable microcatheter (LEONIS Mova; Sumitomo Bakelite, Tokyo, Japan). The steerable microcatheter was passed through the shunt route without creating an angle. Digital subtraction venography (DSV) was performed to assess and confirm GV. We planned to place the coil behind the left inferior pulmonary vein. The steerable catheter was inverted from distal to the coil embolization site (Fig. 2a). Appropriately sized detachable coils (Target XL and XXL, Stryker:16, AZUL CX18, Terumo:1) were deployed using a 1.9 Fr micro catheter (Carnelian MARVEL; Tokai Medical Products, Aichi, Japan) through the inverted steerable catheter. Complete coil occlusion of the outflow shunt was confirmed, and the inferior phrenic vein was not visualized (Fig. 2b). Next, after moving the tip of microcatheter to the varices, 5% ethanolamine oleate with iopamidol (EOI, total 24 ml) was injected gradually through the microcatheter to embolize up to the coil in the outflow shunt, varices, and other minor collaterals (Fig. 2c). After waiting sufficiently after each embolization, we confirmed stagnation of the EOI and removed the system. One week after the procedure, contrast-enhanced CT was performed, which confirmed complete occlusion of GV (Fig. 2d). No complications were encountered.

## Discussion

We were unable to introduce a balloon catheter in the present case because of mild stenosis between the IVC and the left inferior phrenic vein and its shunt angle. We did not consider a venoplasty of the stenosis because of the risk of rupture. The technique of CARTO is considered to be an effective alternative technique in cases where BRTO is difficult due to shunt size, shunt angle, or vessel tortuosity (Kobayakawa et al. 2019; Lee et al. 2014) . However, CARTO requires multiple coils to achieve occlusion of the shunt vein. For this reason, CARTO is expected to have higher material costs than common BRTO, which is a disadvantage of the technique compared with BRTO.

Regarding the sclerosing agent, in the first reported procedure of CARTO, the authors used gelfoam to embolize and reduced the risk of previously reported serious complications associated with BRTO by using EOI (Lee et al. 2014). We considered that gelfoam might not achieve complete occlusion in the long term, because it only provides temporary embolization. In a retrospective study, plug-assisted retrograde transvenous obliteration using gelfoam was found to be associated with frequent recurrence of GV, in contrast to BRTO with EOI or sodium tetradecyl sulfate (STS) foam (Kim et al. 2016). In the present case, we used 5% EOI, which resulted in no complications including pulmonary embolism, portal vein thrombosis, or renal dysfunction. Liquid sclerosants could be used to safely confirm the stasis using stepwise injection. Various sclerosants such as EOI, STS, polidocanol, and gelatin sponge are used in BRTO and modified BRTO (Kobayakawa et al. 2019), but identification of an optimal sclerosant requires further study.

**Fig. 3:**
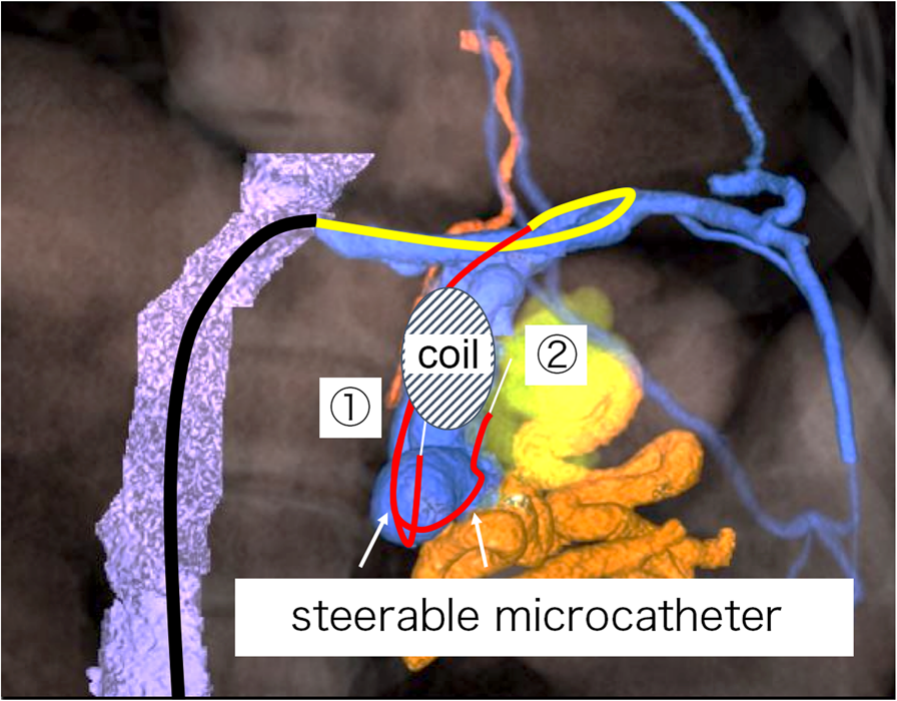
The schema of coil-assisted retrograde transvenous obliteration from a single access route using a steerable microcatheter. The procedure was carried out using the following steps: ① The steerable catheter was inverted from distal to the coil embolization site and detachable coils deployed through the inverted steerable catheter using a microcatheter; ② Sclerosing agent such as 5% ethanolamine oleate with iopamidol was injected from the microcatheter through the steerable catheter

Lee et al. reported the use of two microcatheter systems from double access routes for CARTO (Lee et al. 2014). One micro-catheter is used to release the detachable coil, and the other for injecting the sclerosing agent. In the present case, inverting the steerable microcatheter enabled the coil to be deployed and the EOI injected from a single access route (Fig. 3). The use of a steerable microcatheter is a novel approach which appears to be useful in CARTO to reduce the required access routes.

## Conclusions

In conclusion, CARTO has advantages over BRTO in cases where performing BRTO is difficult, while CARTO is more expensive than BRTO. Using a steerable microcatheter simplifies the procedure by reducing the required access routes in CARTO.

## Data Availability

The datasets used and/or analysed during the current study are available from the corresponding author on reasonable request.

## Abbreviations

BRTO: Balloon-occluded retrograde transvenous obliteration
CARO: Coil-assisted retrograde transvenous obliteration
CT: Computed tomography
CTAP: Computed tomography during arterial portography
DSV: Digital subtraction venography
EOI: Ethanolamine oleate with iopamidol
GV: Gastric varices
HCC: Hepatocellular carcinoma
IVC: Inferior vena cava
STS: Sodium tetradecyl sulfate
